# Modulation of the Wound Healing through Noncoding RNA Interplay and GSK-3*β*/NF-*κ*B Signaling Interaction

**DOI:** 10.1155/2021/9709290

**Published:** 2021-08-26

**Authors:** Xianyi Wang, Wanqiu Li, Shengdi Lu, Zhongliang Ma

**Affiliations:** ^1^Lab for Noncoding RNA & Cancer, School of Life Sciences, Shanghai University, Shanghai 200444, China; ^2^Department of Orthopedic Surgery, Shanghai Jiao Tong University Affiliated Sixth People's Hospital, 600 Yishan Road, Xuhui District, Shanghai 200233, China

## Abstract

Diabetic foot ulcers are seriously endangering the physical and mental health of patients. Due to the long duration of inflammation, the treatment of nonhealing wounds in diabetes is one of the most prominent healthcare problems in the world. The nuclear factor kappa B (NF-*κ*B) signaling pathway, a classical pathway that triggers inflammatory response, is regulated by many regulators, such as glycogen synthase kinase 3 beta (GSK-3*β*). Noncoding RNAs, a large class of molecules that regulate gene expression at the posttranscriptional or posttranslational level, play an important role in various stages of wound healing, especially in the stage of inflammation. Herein, we summarized the roles of noncoding RNAs in the NF-*κ*B/GSK-3*β* signaling, which might provide new ideas for the treatment of diabetic wound healing.

## 1. Introduction

Diabetes is putting abound of negative influence on human health. The report of International Diabetes Federation (IDF) in 2017 illustrated that there were 451 million adult patients with diabetes worldwide, and by 2045, this number would increase to 693 million [1]. Recent studies have shown that the inflammatory response not only plays a significant role in the occurrence and development of type 2 diabetes mellitus, but also in its complications [2]. Diabetic foot ulcer (DFU) is one of the major complications of this disease, which results in a dramatic decline in the ability of wound healing [3].

RNAs have long been considered to act as messengers in the gene expression, according to the central dogma. For example, mRNAs are mainly involved in the information transmission between DNAs and proteins [4]. However, increasing studies have shown that most regions in the human genome are noncoding. The noncoding RNAs transcribed from these regions do not encode proteins. Instead, they regulate the gene expression at the posttranscriptional or posttranslational level, which greatly enriches the connotation of the central dogma [5].

NF-*κ*B signaling is a crucial pathway in the inflammatory response [6]. When immune responses initiate, it will be transferred to the nucleus as a transcription factor and promotes intracellular inflammation. GSK-3*β*, an indispensable molecule regulating the NF-*κ*B signaling pathway, is associated with glucose metabolism, cell differentiation, proliferation, and inflammatory reaction [7]. The development of DFU is related to a variety of noncoding RNAs, such as microRNAs (miRNAs) [8], long noncoding RNAs (lncRNAs) [9], circular RNAs (circRNAs) [10], and tRNA-derived fragments (tRFs), which participate in the GSK-3*β*/NF-*κ*B pathway.

In this review, we elaborated the mechanism of noncoding RNAs, especially miRNAs, lncRNAs, circRNAs, and tRFs in the diabetic wound mediated by the NF-*κ*B/GSK-3*β* signaling.

## 2. Diabetic Wound Healing

Diabetic wound healing can be divided into three periods: (1) inflammatory reaction: various inflammatory cells infiltrate and secrete a large number of cytokines and chemokines, through which apoptotic cells and necrotic tissue can be eliminated; (2) proliferation: in this stage, the proliferation of granulation tissue and capillary is initiated; and (3) tissue reconstruction: capillary degeneration, extracellular matrix recombination, and further maturation of new tissue mainly occur during this period [11].

The formation of diabetic foot ulcer is closely related to the metabolic disorder of diabetes. Foot diseases caused by diabetes include

1. Infections, which are mainly induced by the mold and bacterial invasion under the continuously increased blood sugar
2. Ulcer, defects caused by the destruction of the dermis or deep layers of the skin, which usually occurs after a skin infection
3. Damage to the foot tissue, which happens in nearly 6% of the patients.

These diseases affect patients not only in their quality of life but also in their social life and livelihoods [12]. In addition, studies have shown that 0.03% to 1.5% of patients with diabetic foot need to be amputated [13].

Compared with normal healing process, the inflammatory stage of diabetic foot ulcer wound is abnormally prolonged, making the ulcer wound difficult to heal and easy to recur. Therefore, it is necessary for us to research the inflammatory mechanism of diabetic foot.

## 3. NF-*κ*B Signaling

The coordinated activation of multiple signaling pathways is the character4istic of inflammatory response. Proinflammatory and anti-inflammatory factors were recruited in this stage [14]. The NF-*κ*B signaling is a typical proinflammatory pathway. The roles of NF-*κ*B in the expression of cytokines, chemokines, adhesion molecules, and other proinflammatory factors have been studied extensively [15].

NF-*κ*B consists of five main components, p50, RelA(p65), RelB, p52, and Rel(c-Rel), which could form p50/p65 dimer and p52/RelB dimer [16]. All these subunits contain a common domain, the Rel homologous domain (RHD). RHD is composed of 300 amino acids, which mediates the DNA binding, inhibitory protein binding, and dimerization through sequence specificity [17].

Three different modes are involved in the activation of NF-*κ*B [18]. In the canonical NF-*κ*B activation pathway, the with NF-*κ*B inhibitor (I*κ*B) being phosphorylated and degraded, the NF-*κ*B dimer (p65/p50) will enter the nucleus, acting as a transcriptional activator [19]. Toll-like receptor-4 (TLR-4) has been proved to stimulate the activation of NF-*κ*B once it binds with lipopolysaccharide (LPS) [20].

The noncanonical activation pathway is mainly mediated by the constitutive processing of p100 to p52 [21]. Under the inactivated state, this pathway is suppressed by the tumor necrosis factor (TNF) receptor-associated factors 2 and 3(TRAF2 and TRAF3) [22]. Compared with the canonical activation of the NF-*κ*B pathway, the NF-*κ*B molecules have limited ligands and a lower activation rate in the noncanonical activation pathway [23].

The CBM (CARMA1, BCL10, and MALT1) complex contributes greatly to the NF-*κ*B pathway as well. The NF-*κ*B activation mediated by CARMA1 is closely associated with G protein-coupled receptor (GPCR) superfamily binding [24]. MALT1 is a downstream molecule which could act as a scaffold or protease for NF-*κ*B activation [25]. A growing number of studies have shown that the CBM-regulated activation pathway is crucial in inflammation and tumorigenesis [26].

## 4. GSK-3*β*/NF-*κ*B Signaling in Diabetic Wound Healing

GSK dysregulation is quite vital in the pathogenesis of inflammation-induced disorders, affecting the development of diabetic wound healing [27], diabetic nephropathy [28], and diabetic retinopathy [29]. NF-*κ*B is a common downstream molecule involved in several GSK-related pathways associated with these diseases.

There are two main subtypes of GSK, GSK-3*α*, and GSK-3*β* [30]. GSK-3*β* is a serine/threonine kinase [31], as a negative regulator of glucose homeostasis; it participates in many physiological and pathological processes, such as energy metabolism [32] and inflammation progression [33, 34]. In addition, recent research indicated that GSK-3*β* could control tumorigenesis by inhibiting the autophagy pathways [35]. The phosphorylation of Ser9 at the amino terminal of GSK-3*β* could significantly inhibit its activity, while the phosphorylation of Tyr216 could promote its activity [36]. Therefore, different phosphorylation sites will have different effects.

There are more and more signaling pathways related to inflammation with GSK-3*β* involvement being discovered, such as the GSK-3*β*/Wnt/*β*-catenin/NF-*κ*B axis [37] and AKT/GSK-3*β*-Nrf2/NF-*κ*B axis [38]. With the deepening of understanding, the fact that oxidative stress and the abnormal activation of inflammation are inseparable has been widely recognized [39, 40]. Zhang and his colleagues found that the expression levels of p-Akt, p-GSK-3*β*, p-Nrf2, and HO-1 were rapidly decreased in nephrectomized rats. However, when the kidney injury and inflammation were attenuated, these protein levels recovered again, suggesting that the GSK-3*β*/NF-*κ*B signaling pathway could make a difference in the inflammation progression [41]. Suppressing the expression of GSK-3*β* could also activate AMP-activated protein kinase (AMPK), thus reducing proinflammatory responses and enhancing anti-inflammatory immune responses in livers [42]. Additionally, this inhibitory action could inhibit the transcriptional activity of NF-*κ*B which in turn enhances the transcriptional activity of cAMP response element binding protein (CREB1). This resulted in the slowing down of the inflammatory reaction caused by liver injury and inhibit the apoptosis of hepatocytes [42].

Although the role of GSK-3*β*/NF-*κ*B axis has not been fully elucidated in angiogenesis, the involvement of NF-*κ*B in the process of angiogenesis has been well interpreted. The p65 subunit of NF-*κ*B acts directly on the promoter region of the angiogenic factor with G-patch and FHA domains 1 (AGGF1), a key molecule in angiogenesis, promoting angiogenesis [43]. The elevated NF-*κ*B expression stimulates the recruitment and M1 polarization of macrophages, which attenuates the progression of angiogenesis [44]. More and more mechanisms NF-*κ*B involved are being discovered, opening a new chapter for NF-*κ*B functions research.

GSK-3*β* also performs irreplaceably in angiogenesis, affecting the progress of diabetic wound healing. GSK-3*β* induced the degradation of *β*-catenin, a key factor for keratinocyte proliferation and migration, through *β*-catenin phosphorylation and destabilization [45, 46]. Treating keratinocyte HaCaT with lucidone could promote its proliferation, migration, and invasion, which are accompanied with the increased expression level of *β*-catenin. What is more, a decreased GSK-3*β* expression was observed in lucidone treated sample, suggesting that the increased angiogenesis is inhibited by GSK-3*β* [47]. Similarly, an increased Nrf2 but decreased ROS expression was detected in angiogenesis, which points to a key role of the GSK-3*β*/Nrf2 axis in suppressing angiogenesis [48].

Moreover, the key roles of GSK-3*β* in DNA repair, apoptosis, and cancer treatment make it a reliable therapeutic target [49]. What is more excited is that GSK inhibitors have shown unique application prospects in DFU therapy for its favorable effects of anti-inflammatory and proangiogenesis.

## 5. miRNAs and Wound Healing Progression

miRNAs are small endogenous noncoding RNAs (18–25 nucleotides) that exert their effects via posttranscriptional regulation [50]. miRNAs are involved in the whole stages of wound healing (Table 1). In the stage of proliferation, miR-15b and miR-200b targets the 3′-UTR of vascular endothelial growth factor (VEGF) and VEGFR2, respectively, blocking the pathways they participate in, thereby inhibiting angiogenesis [51]. On the contrary, miR-205 can directly target the phosphatase and tensin homolog (PTEN) and promote the activation of the Akt pathway, thus promoting angiogenesis and endothelial cell proliferation [52]. In the stage of tissue remodeling, miR-129 and miR-335 can downregulate Sp-1, reduce MMP-9 further, and subsequently promote the deposition of extracellular matrix [53]. Collagen deposition is particularly important for wound tissue reconstruction. Britta et al. found that miR-29 could target collagen gene directly, which makes miR-29 an important regulator in the tissue reconstruction on the wound surface [54].

**Figure 1:**
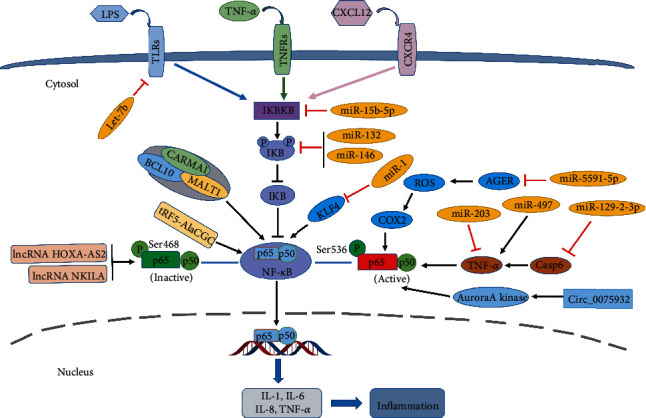
Noncoding RNAs and NF-*κ*B signaling pathway. Lipopolysaccharide (LPS), tumor necrosis factor-*α* (TNF-*α*), and C-X-C motif chemokine ligand 12 (CXCL12) can activate the NF-*κ*B signaling pathway via the upregulation of I*κ*B kinase (IKK). The noncoding RNAs that stimulate or inhibit the activation and cytoplasm-nucleus transport of NF-*κ*B are shown in the figure. Consequently, these noncoding RNAs can be classified into proinflammatory noncoding RNAs and anti-inflammatory noncoding RNAs, according to their roles in the NF-*κ*B signaling pathway.

It is worth noting that the inflammatory response is the first stage of wound healing, and its regulatory mechanism is particularly critical to the process of wound healing. Therefore, several miRNAs related to the inflammatory response will be expounded in this review (Figure 1).

### 5.1. GSK-3*β*/NF-*κ*B-Related miRNAs in Inflammation

**Figure 2:**
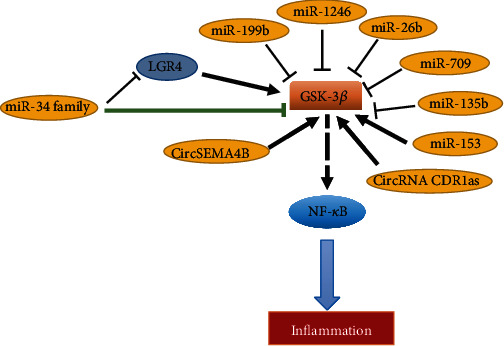
The role of GSK-3*β*/NF-*κ*B signaling in inflammation. The miR-34 family could target GSK-3*β* directly and may be closely correlated with NF-*κ*B, a molecule that is crucial in the inflammatory stage of diabetic wound healing. The green arrowhead in figure indicates that this regulatory interaction is being proven.

Multiple studies found that miRNAs can regulate GSK activity (Figure 2), thereby affecting the occurrence and development of inflammation, which in turn exert influences on neurological diseases such as Parkinson's disease (PD) and Alzheimer's disease (AD) [55]. In addition, grape seed procyanidin extract (GSPE) was found protecting against lead-induced liver injury via decreasing the expression of miR-153. As a result, the translocation of Nrf2 and the phosphorylation of GSK-3*β* at Ser9 increased a lot [56]. Acute rejection after organ transplantation is mainly associated with inflammatory response [57]. Restraining the activation of GSK-3*β*, NF-*κ*B, and several inflammatory cytokines by the transfection of miR-199b-5p mimic would attenuate the inflammatory response significantly and might provide a new solution to the rejection after lung transplantation [58].

**Figure 3:**
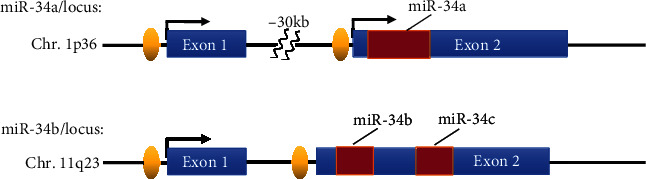
Structure of genomic loci of the human miR-34 family. The miR-34 family consists of 3 members, miR-34a, miR-34b, and miR-34c, which share different genomic loci in chromosomes. This figure shows the distribution of miR-34 family on chromosomes.

One of the most well-studied miRNAs in inflammation related to GSK-3*β* is the miR-34 family. miR-34 family can be divided into three members, miR-34a, miR-34b, and miR-34c [59]. miR-34a has a unique transcript on chromosome 1, while miR-34b and miR-34c share one transcript whose gene loci are on chromosome 11 [60] (Figure 3). A recent study revealed that miR-34 family might act as a proinflammatory factor in the wound healing process of venous ulcers patients. Wu et al. found that miR-34a and miR-34c were significantly upregulated on the surface of venous ulceration wounds, compared with other parts of the skin or normal wound sites [61]. Luciferase reporter assay revealed that miR-34a could directly target leucine rich repeat containing G protein-coupled receptor 4 (LGR4). What is more, the quantitative real-time polymerase chain reaction (qRT-PCR) and enzyme-linked immunosorbent assay (ELISA) indicated that the NF-*κ*B signaling pathway was abnormally activated under the status of miR-34a/c overexpression. The expression of GSK-3*β* was also detected in the skin of LGR4 KO and WT mice, through which an abnormal activation of GSK-3*β* was existed in LGR4 deficient mice [61].

However, GSK-3*β* is a versatile kinase which is manifested in its two-faced nature in inflammation regulation [62]. Contrary to most known cognitions, inhibition of GSK-3*β* also reduces the termination of inflammation, which is resulted from the reduced autophagy or apoptosis and retarded resolution of inflammation [63, 64]. Accordingly, elucidating the inhibitory effects of miRNAs on GSK-3*β* is of great significance for the treatment of inflammation. In the acute inflammation models of mouse liver and cell lines, researchers investigated that miR-155 was induced by LPS. MiR-155 could inhibit the activity of GSK-3*β*, while the NF-*κ*B signaling was still being activated [65]. This phenomenon is in contradiction with the proinflammatory effects of GSK-3*β* that we usually recognize. Although in recent years, few studies have been published on GSK-3*β* regulated directly by miRNAs in diabetic wound healing, immediate relationships have been studied in other diseases. For examples, miR-29a inhibited the expression of GSK-3*β* by binding to the 3′-UTR of its mRNA and forming the RNA-induced silencing complex (RISC), thereby ameliorating the nonalcoholic steatohepatitis in mice [66]. Interestingly, in human colorectal cancer (hCRC), miR-377-3p upregulated the expression of GSK-3*β* by targeting its 3′-UTR in a motif-dependent manner, which is contrary to our well-known mechanism of miRNA action, i.e., targeted inhibition [67]. In addition, miRNAs are also involved in the cancer stem cell generation, epithelial to mesenchymal transition, invasion, and metastasis via regulating the signaling pathway of GSK-3*β* participation [68].

Our previous results confirmed that GSK-3*β* is a direct target for miR-34 family. We hypothesized that miR-34a might be involved in the progression of diabetic wound healing by targeting GSK-3*β* directly. Still, the effects of miR-34a on the inflammatory stage of diabetic wound healing through GSK-3*β* remain to be explored. We believe that GSK-3*β* will become another reliable target in the treatment of diabetic wound healing.

### 5.2. Proinflammatory miRNAs

miRNAs can be divided into proinflammatory miRNAs and anti-inflammatory miRNAs, according to their roles in the signal transduction pathway. miR-1 carried by extracellular vesicles derived from steatotic hepatocyte can promote endothelial inflammation and atherogenesis. Such proinflammatory effect is mediated by an anti-inflammatory factor, Kruppel-like factor 4 (KLF4). Under inflammatory state, the sustained KLF4 expression may interact with p300, a coactivator required for activation of NF-*κ*B, and sequester it away from NF-*κ*B. In this way, the expression of NF-*κ*B is greatly decreased, and the inflammatory level is reduced [69]. However, the binding between miR-1 and 3′-UTR of KLF4 restrained the anti-inflammatory effect of KLF4 and stimulate the inflammatory response in cells [70].

Bala et al. found that alcohol-induced steatohepatitis was mitigated significantly in miR-155 knockout mouse models. This inhibitory effect was largely due to the decreased lipid metabolism-related genes and the repressed CD163(+), CD206(+) infiltrating macrophages and neutrophils [71].

Inflammatory M1 spectrum macrophages protect the body from infection but cause inflammatory diseases and damage to the tissues, while activating M2 spectrum macrophages reduces inflammation and promotes tissue repairing [72]. Jablonski et al. found that miR-155 was significantly upregulated in inflammatory M1 (LPS+IFN-*γ*) but not in inflammatory M2 (IL-4) macrophages. The expression of genes related to M1 macrophages, *iNOS*, *IL-1b*, and *TNF-a*, was reduced by 72% in miR-155 knockout mouse, while miR-155 deficiency did not affect the expression of M2-related gene *Arg1* [73]. The above experimental results suggest that miR-155 functions as a proinflammatory regulator in the inflammatory response.

### 5.3. Anti-Inflammatory miRNAs

Anti-inflammatory miRNAs are of great therapeutic significance for the treatment of diseases caused by excessive inflammation. F-box and WD repeat domain-containing protein 7 (FBW7), a class of Skp1–Cullin–F-box-protein (SCF) ubiquitin ligase, have been proved to be involved in the pathogenesis of hematological tumors [74], non-small cell lung cancer [75], and cardiac diseases [76]. Meng et al. first studied the function of FBW7 on inflammatory bowel disease and identified its upstream controller, miR-129. They found FBW7 could upregulate NF-*κ*B by inducing the ubiquitin-dependent degradation of I*κ*B*α*. miR-129 could lower the expression of FBW7, leading to the inhibition of the NF-*κ*B pathway and amelioration of intestinal inflammation [77].

miR-497 is another classical anti-inflammatory miRNA. Eunmi et al. found that the process of wound healing was accelerated significantly after the miR-497 mimics were injected subcutaneously to the wound tissue of diabetic rats. With the overexpression of miR-497, the proinflammatory factors in the wound, IL-1*β*, IL-6, and TNF-*α*, were decreased significantly [78].

Our lab has elaborated the function of miR-296-5p in the diabetic wound healing. We found that miR-296-5p acted as a suppressor in this progress with cell proliferation and cell cycle inhibition. To further elucidate the molecular mechanisms of miR-296-5p, the *sodium-dependent glucose carrier 2 (SGLT2)*, a gene mainly that mediates the reabsorption of glucose, was identified as a potential target of miR-296-5p. The experiments in vivo indicated that miR-296-5p agomiR promoted the healing of diabetic wounds in the wounded diabetic rat model significantly. All these phenomena revealed that miR-296-5p not only can be used as a diagnostic marker of tumor but also accelerates the healing of diabetic wounds. In brief, miR-296-5p might be an effective molecular tool for the diagnosis and treatment of diabetes [7].

The coordination failure between the immune system and the microbiome combined with the presence of infection is the main factor that triggers the “cytokine storm.” That is, in some cases, certain microbes can cooperate with pathogens to provide favorable conditions for pathogen invasion [79]. *Staphylococcus aureus* exists in the tissues of patients with diabetic foot ulcer [80]. Ramirez et al. found that in the diabetic foot ulcer model, the expression of miR-15b-5p induced by *S. aureus* increased significantly. It participates in the damage repair of DNA and deregulates inflammatory response by targeting the downstream WEE1 and IKBKB [81].

Therefore, from a diagnostic point of view, anti-inflammatory miRNAs may be used as good treatments to benefit the patients with diabetic foot ulcer [82].

## 6. lncRNAs, circRNAs, and tRFs in GSK-3*β*/NF-*κ*B Signaling

lncRNAs are more than 200 nucleotides in length and transcribed by RNA Pol II. They are capable of regulating gene expression at epigenetic, transcriptional, and posttranscriptional levels [83, 84]. Many studies suggest that lncRNAs could regulate GSK-3*β* activity directly or indirectly (i.e., by interacting with miRNA). lncRNA Rik-203 can increase neural differentiation and reduce the neurotoxicity of anesthetics by promoting the expression of GSK-3*β* [85]. In hepatocellular carcinoma, knockdown of lncRNA IHS reduced the level of phosphorylated GSK-3*β*, which greatly inhibited the activity of AKT/GSK-3*β* signaling pathway, and promoted the proliferation and metastasis of tumors [86]. As mentioned earlier, GSK-3*β* can regulate the expression of NF-*κ*B, thereby altering the inflammatory state in vivo. In addition, some researches indicated that lncRNAs could regulate the function of NF-*κ*B via various modes of action [87, 88]. Still, whether lncRNAs can regulate the NF-*κ*B signaling pathway by affecting GSK-3*β* activity and their roles in DFU is worthy of further investigation.

lncRNAs can be competing endogenous RNAs (ceRNAs) of miRNAs and serve as their “sponge” to suppress the function of miRNAs [89]. The wound healing ability in DFU patients was greatly enhanced by incubating fibroblasts with lncRNA H19-transfected mesenchymal stem cell [90]. The underlying mechanism is that lncRNA H19 acted as the “sponge” of miR-152-3p, thus promoting the expression of the miRNA target gene, PTEN. This competitive effect activated the PI3K/AKT signaling pathway, thereby inhibiting the inflammatory response and apoptosis of fibroblasts, and eventually promoted the wound healing ability of type 2 diabetes mellitus mice [90]. Furthermore, many studies asserted that a strong interaction was existed between the lncRNAs and NF-*κ*B signaling pathways. For instance, lncRNA HOXA-AS2 inhibited the activity of NF-*κ*B through a negative feedback loop to repress endothelium inflammation [91]; lncRNA NKILA overexpression inhibited the phosphorylation of IKB and nuclear translocation of the p65 subunit, thus suppressing the expression of proinflammatory cytokines [92].

circRNAs, a class of RNAs covalently linked at the 3′ and 5′ ends, are derived from the variable splicing of RNA precursors [93]. They regulate gene expressions in three main ways: 1 sponge miRNAs, which is consistent with the ways lncRNAs work; (2) change the splicing patterns and the stabilities of mRNAs through binding with mRNA-binding proteins (RBPs); and (3) some circRNAs containing open reading frames (ORFs) can encode proteins [94]. The expression profiles of various circRNAs have undergone significant changes in DFU. Hsa_circ_0084443 was proved to be upregulated in DFU, which exhibited reduced motility and enhanced growth rate of the keratinocytes [10]. circRNAs usually regulate the activation of GSK-3*β*/NF-*κ*B signaling in the manner of miRNA sponges. circSEMA4B promoted the expression of GSK-3*β* by acting as the sponge of miR-431 and inhibited the degenerative process induced by IL-1*β* in nucleus pulposus cells [95]. Exendin-4 reversed the PC12 cells damage induced by 1-methyl-4 phenyl-pyridine ion (MPP^+^), and this effect was largely achieved by inhibiting the expression of circRNA CDR1as. circRNA CDR1as sponged miR-671 and released the expression of GSK-3*β*, which was demonstrated experimentally by dual luciferase reporter assay. The increased GSK-3*β* phosphorylation and activated PI3K/AKT/GSK-3*β* pathway induced the damage of PC12 cells and the occurrence of Parkinson's disease [96]. circRNAs also regulate the inflammation through the NF-*κ*B pathway. Exosome derived from circ_0075932-overexpressed adipocytes facilitated the apoptosis and inflammation of dermal keratinocytes remarkably. circ_0075932 could bind with RNA-binding protein, PUM2, which was reported to stimulate the expression of Aurora-A kinase, thus activating the NF-*κ*B pathway [97]. Therefore, circRNAs take important parts in the progress of inflammation and wound healing.

In addition to miRNAs, lncRNAs, and circRNAs, tRFs can interact with NF-*κ*B as well. Therefore, tRFs may have potential application prospects in the treatment of DFU wound healing. Although the action mechanism of tRFs has not yet been fully elucidated, their roles as a class of small noncoding RNAs involved in the occurrence and development of disease have received widespread attention. Liu et al. found that in heavy metal-induced cellular responses, the expression of tRF5-AlaCGC derived from the mature tRNA-AlaCGC 5′ end increased significantly, and downregulation of tRF5-AlaCGC would inhibit the nuclear translocation of p65, thus limiting the activity of NF-*κ*B [98]. This suggests that tRFs have important implications in the inflammatory response.

## 7. Conclusion and Future Perspectives

One of the most notable characteristics of diabetic foot ulcer is that the chronic inflammatory reaction starts late, lasts long, and is difficult to subside [99]. NF-*κ*B, as an important nuclear transcription factor, participates in the inflammatory stage of the wound healing process by inducing the production of proinflammatory factors [6, 100]. Therefore, the inflammatory reaction of wound healing can be manipulated by regulating the factors related to the NF-*κ*B signaling pathway. In this way, the wound healing efficiency of patients with diabetic foot ulcer can be improved effectively.

As an effective regulator, miRNA is essential to the process of wound healing. It has become a new therapeutic method and target due to its diverse functions [101, 102]. Additionally, the therapeutic effects of synthetic miRNA mimics or inhibitors have been verified in the animal experiments. Several miRNA therapies that entered the clinical trial stage have shown low toxicity and effective target organ delivery. However, there is still some uncertainty despite the advantages mentioned above, for example, it is not clear whether the nonphysiological concentration of miRNA during treatment will cause nonspecific of targets or disrupt the imbalance of cell homeostasis [103]. Therefore, it is necessary for us to research the expression profile and the target genes of miRNAs to ensure their safety.

GSK-3*β* is vital in the process of inflammatory and angiogenesis. However, as we mentioned earlier, the direct relationship between GSK-3*β* and NF-*κ*B in angiogenesis remains unclear. We hypothesize that GSK-3*β* may modulate NF-*κ*B through phosphorylation, which follows the action mechanism of GSK-3*β*. Still, there is sufficient evidence to confirm that GSK-3*β* regulates NF-*κ*B indirectly through other pathways, like the *β*-catenin signaling. In the consequent work, in order to seek suitable treatment targets for diabetic wound healing, we need to explore the direct targeting relationship between specific miRNAs and GSK-3*β*, seek the immediate connection between GSK-3*β* and NF-*κ*B in angiogenesis, and give full play to their effects in the wound healing process. In addition, another fact worth noting is that tRFs have the same targeted silencing mechanism as miRNAs [104]. Therefore, studying whether tRFs play inflammatory or anti-inflammatory roles in the NF-*κ*B signaling pathway is another research direction, and this may open a new chapter for the diabetic wound healing treatment.

## Figures and Tables

**Table 1:** Noncoding RNAs and their targets in wound healing.

Noncoding RNA	Action stage	Control effects	Targets
miR-34a	Inflammation	↑	LGR
miR-1	Inflammation	↑	KLF4
miR-155	Inflammation	↑	Bcl6
miR-129	Inflammation	↑	FBW7
miR-156-5p	Inflammation	↑	WEE1, IKBKB
miR-497	Inflammation	↓	IRAK2
miR-16	Inflammation	↓	COX2
miR-129-2-3p	Inflammation	↓	Casp6, Ccr2
tRF-AlaCGC	Inflammation	↑	NF-*κ*B
H19	Inflammation	↓	miR-152-3p
HOXA-AS2	Inflammation	↓	NF-*κ*B
NKILA	Inflammation	↓	NF-*κ*B
circSEMA4B	Inflammation	↓	miR-431
circRNA CDR1as	Inflammation	↑	miR-671
miR-205	Proliferation	↑	PTEN
miR-21	Proliferation	↑	PTEN, RECK
miR-125a	Proliferation	↑	DLL4
miR-148b	Proliferation	↑	TGFB2, SMAD2
miR-126	Proliferation	↑	SPRED1
miR-15b	Proliferation	↓	VEGF
miR-451	Proliferation	↓	MIF
miR-200b	Proliferation	↓	VEGFR2
circ_0084443	Proliferation	↑	
miR-129/miR-335	Reconstruction	↑	Sp-1
miR-29a	Reconstruction	↓	TAB-1
miR-198	Reconstruction	↓	DIAPH1, PLAU, LAMC2
miR-191	Reconstruction	↓	SATB1
